# Allelic losses in carcinoma in situ and testicular germ cell tumours of adolescents and adults: evidence suggestive of the linear progression model

**DOI:** 10.1054/bjoc.2000.1334

**Published:** 2000-08-17

**Authors:** S W Faulkner, D A Leigh, J W Oosterhuis, H Roelofs, L H J Looijenga, M L Friedlander

**Affiliations:** Molecular and Cytogenetics Unit, Department of Haematology, SEALS, Prince of Wales Hospital, High St, Randwick, NSW, 2031, Australia; Laboratory for Experimental Patho-Oncology/Pathology, Daniel den Hoed Cancer Centre, Josephine Nefkens Institute, FGG/EUR, Building Be, PO Box 1738, Rotterdam, 3000DR, The Netherlands; Department of Medical Oncology, Prince of Wales Hospital, High St, Randwick, NSW, 2031, Australia

**Keywords:** loss of heterozygosity, carcinoma in situ, testicular germ cell tumours

## Abstract

Testicular germ cell tumours (TGCTs) may arise through a process of multi-step carcinogenesis, and loss of heterozygosity (LOH) at specific loci is likely to be an important early event, although this has not been studied in detail. In order to explore the pathogenetic relationships among TGCTs, we investigated the genetic changes in testicular tumours that exhibit a disease continuum through the precursor carcinoma in situ (CIS) to either seminoma (SE) and/or non-seminomatous germ cell tumour (NSGCT). Universal amplification has been performed on 87 TGCT specimens and 36 samples of CIS cells microdissected from single paraffin-embedded tumour sections from 40 patients, including multiple specimens of CIS and TGCT cells of varied histology microdissected from 24 individual patients. Seventy-seven microsatellite markers were used to assay these samples for LOH at candidate regions selected from the literature, mapping to 3q, 5q, 9p, 11p, 11q, 12q, 17p and 18q. Construction of deletion maps for each of these regions identified common sites of deletion at 3q27–q28, 5q31, 5q34–q35, 9p22–p21 and 12q22, which correlate with allelic losses we have also observed in the precursor CIS cells. Evidence for allelic loss at 3q27–q28 was observed in all of the embryonal carcinoma samples analysed. We conclude that inactivation of gene(s) within these regions are likely to be early events in the development and progression of TGCTs. These results also provide molecular evidence in support of the hypothesis that SE is an intermediate stage of development within a single neoplastic pathway of progression from CIS precursor cells to NSGCT. © 2000 Cancer Research Campaign


*British Journal of Cancer* (2000) **83**(6), 729–736
© 2000 Cancer Research Campaign
doi: 10.1054/ bjoc.2000.1334, available online at http://www.idealibrary.com on IDEAL®

### Allelic losses in carcinoma in situ and testicular germ cell tumours of adolescents and adults: evidence suggestive of the linear progression model

**SW Faulkner[1], DA Leigh[1], JW Oosterhuis[2], H Roelofs[2], LHJ Looijenga[2] and ML Friedlander[3]**

[1]Molecular and Cytogenetics Unit, Department of Haematology, SEALS, Prince of Wales Hospital, High St, Randwick, NSW 2031, Australia; [2]Laboratory for Experimental Patho-Oncology/Pathology, Daniel den Hoed Cancer Centre, Josephine Nefkens Institute, FGG/EUR, Building Be, PO Box 1738, 3000DR, Rotterdam, The Netherlands; [3]Department of Medical Oncology, Prince of Wales Hospital, High St, Randwick, NSW 2031, Australia

**Summary** Testicular germ cell tumours (TGCTs) may arise through a process of multi-step carcinogenesis, and loss of heterozygosity (LOH) at specific loci is likely to be an important early event, although this has not been studied in detail. In order to explore the pathogenetic relationships among TGCTs, we investigated the genetic changes in testicular tumours that exhibit a disease continuum through the precursor carcinoma in situ (CIS) to either seminoma (SE) and/or non-seminomatous germ cell tumour (NSGCT). Universal amplification has been performed on 87 TGCT specimens and 36 samples of CIS cells microdissected from single paraffin-embedded tumour sections from 40 patients, including multiple specimens of CIS and TGCT cells of varied histology microdissected from 24 individual patients. Seventy-seven microsatellite markers were used to assay these samples for LOH at candidate regions selected from the literature, mapping to 3q, 5q, 9p, 11p, 11q, 12q, 17p and 18q. Construction of deletion maps for each of these regions identified common sites of deletion at 3q27–q28, 5q31, 5q34–q35, 9p22–p21 and 12q22, which correlate with allelic losses we have also observed in the precursor CIS cells. Evidence for allelic loss at 3q27–q28 was observed in all of the embryonal carcinoma samples analysed. We conclude that inactivation of gene(s) within these regions are likely to be early events in the development and progression of TGCTs. These results also provide molecular evidence in support of the hypothesis that SE is an intermediate stage of development within a single neoplastic pathway of progression from CIS precursor cells to NSGCT. © 2000 Cancer Research Campaign

**Keywords**: loss of heterozygosity; carcinoma in situ; testicular germ cell tumours

Testicular cancers are the most common malignancy in young adult males. The aetiology of testicular germ cell tumours (TGCTs) of adolescents and adults remains unknown, but certain risk factors such as cryptorchidism (Batata et al, 1982) and carcinoma in situ (CIS) (van der Maase et al, 1986) are known to be important predisposing risk factors. TGCTs are divided into two entities; seminomatous and non-seminomatous germ cell tumours (Mostofi and Sobin, 1977). Seminoma (SE) is composed of cells that are considered the neoplastic counterparts of the primitive germ cells (gonocytes). Non-seminomatous germ cell tumours (NSGCTs) have embryonal carcinoma cells as their stem cell, which can differentiate into extra embryonic (trophoblast and yolk-sac), embryonic (somatic) tissue or a mixture of embryonal carcinoma and both embryonic and extra-embryonic tissues. SE and NSGCT elements may occur simultaneously as combined tumours, or they may be geographically separate or mixed, suggesting that they could share a common developmental pathway via a common precursor. CIS is a neoplastic growth of totipotential cells that, like seminoma, resemble primitive germ cells. It has been proposed that CIS originates from malignant transformation of foetal gonocytes and there is consensus that CIS is the precursor for most testicular germ cell tumours in adults, with the exception of spermatocytic seminoma (Jacobsen et al, 1981; Skakkebæk et al, 1987).

While it is now generally accepted that CIS is the precursor of TGCTs (Jacobsen et al, 1981; Skakkebæk et al, 1987), the developmental relationship between SE and NSGCTs remains poorly understood (Damjanov, 1989). Two main hypotheses have been proposed. The first model is based on the former hypothesis of Pierce and Abell (1970) that assumes SE and NSGCT are independently derived from CIS. According to this theory, the distinct neoplastic pathway for the formation of SE diverges from that of NSGCT at an early stage (Kiss and Jubasz, 1985; Sesterhenn, 1985; Mostofi, 1986). The alternative linear progression model, which is based on the theories of Ewing (1911) and Friedman (1951), assumes that both SE and NSGCTs share a common origin in CIS and develop along a single neoplastic pathway. Seminoma may be either an end-stage in differentiation or an intermediate in the development of NSGCT (Raghavan et al, 1982; Oliver, 1987; Oosterhuis et al, 1989). The linear progression model is further supported by the presence of SE with NSGCT characteristics (Oliver et al, 1987; Czaja and Ulbright, 1992), the finding of NSGCT elements in the metastases of apparently pure primary SE (Bredael et al, 1982) and from DNA-flow cytometry and cytogenetic analysis (Castedo et al, 1989*a*; 1989*b*; Oosterhuis et al, 1989; de Jong et al, 1990; Looijenga et al, 1993; Gillis et al, 1994).

*Received 29 November 1999*
*Revised 23 March 2000*
*Accepted 11 May 2000*

*Correspondence to:* SW Faulkner

In over 85% of cases, the formation of isochromosome 12p, resulting in the over-representation of the short arm of chromosome 12, is the only consistent abnormality noted in TGCTs (Atkin and Baker, 1982). The remaining isochromosome 12p-negative TGCTs invariably exhibit gain of 12p-sequences revealed by fluorescence in situ hybridization (Rodriguez et al, 1993; Suijkerbuik et al, 1993). In addition, about 5% of all TGCTs show specific amplifications corresponding to 12p11.1–12.1 (Mostert et al, 1998). The gene or genes that are over-represented on 12p, and that directly contribute to the development of germ cell tumours, have not yet been identified.

Inactivation of tumour suppressor genes plays a central role in the development and progression of many human cancers. In invasive TGCTs, loss of heterozygosity (LOH) at numerous loci on virtually every chromosome has been observed, making it difficult to ascertain which genetic events are the most crucial to carcinogenesis (Murty et al, 1994; Peng et al, 1995). It is likely that TGCTs arise through a process of multi-step carcinogenesis and that LOH at specific loci is an important early event. Evidence for a minimal region of deletion at 12q22 (Murty et al, 1996*a*) as well as at 5p15, 5q11 and 5q34–q35 (Murty et al, 1996*b*) has been reported, though regions of consistent loss have not yet been defined. It is therefore difficult to determine which, if any, of the genes contained within these regions demonstrating LOH are fundamental to the development of invasive testicular cancer.

To further characterize the pattern of allelic loss in TGCTs it is necessary to investigate specific sub-populations of enriched TGCT and CIS cells for LOH. The use of microsatellite markers for such investigations has been hampered due to limitations and difficulties associated with the PCR amplification of DNA isolated from paraffin-embedded tissue. These difficulties include a high rate of random PCR failure and the poor quality, yield and storage properties of the isolated DNA samples (Shibata et al, 1992; Crisan and Mattson, 1993). This study is the first to investigate the molecular genetic changes in SE and NSGCTs as well as the adjacent CIS cells, using universal genomic amplification as a means of circumventing the technical difficulties. The primer extension pre-amplification (PEP) (Zhang et al, 1992) has been applied to achieve multiple PCR assays on single cells and has recently been shown to be a useful tool for extending limited DNA samples (Casa and Kirkpatrick, 1996). We have modified the existing PEP protocol to generate genomic DNA from sub-populations of enriched TGCT cells and CIS cells microdissected from small regions of paraffin-embedded formalin-fixed tissue sections (Faulkner et al, 1998).

In this study, universal amplification has been applied to specimens of CIS cells and TGCT cells of varying histology microdissected from single paraffin-embedded tumour sections. These included multiple specimens of CIS and TGCT cells of varied histology microdissected from each patient whose tumours represented the pathological continuum observed in TGCTs. This may provide new information about the pathogenetic relationships between these elements of CIS, SE and NSGCT. Several chromosomal regions, including those where consistent LOH in TGCTs has previously been demonstrated, were selected from the literature as candidates for assessment of allele status (Lothe et al, 1993; Murty et al, 1994*a*; 1994*b*; 1996*a*; 1996*b*; Peng et al, 1995; International Testicular Cancer Linkage Consortium, 1998). Based on the pattern of allelic losses, we now define deletion maps of chromosomal regions mapping to 3q, 5q, 9p, 11p, 11q, 12q, 17p and 18q for these pre-amplified CIS and TGCT specimens.

### MATERIALS AND METHODS

### Preparation of DNA from enriched sub-populations of cells

All TGCT specimens were collected at the operating theatre and directly fixed overnight at room temperature in 4% buffered formalin. Consecutive histological sections were cut from paraffin-embedded tumour blocks of these specimens. The first and last sections (4 μm) cut were de-waxed, hydrated, stained with haematoxylin and eosin (H&E) and cover-slipped, while the intermediate section (10 μm) was left unstained and fixed to a microscope slide. Sub-populations of TGCT, CIS or constitutional cells were identified and marked on both H&E-stained slide sections following microscopic examination, ensuring no significant differences were evident between the composition of the marked cellular regions. After careful alignment with the corresponding H&E-stained slide, the selected cellular regions were physically removed from a single unstained section using a scalpel blade. The area of the cells microdissected from a single slide section typically ranged from 1–10 $mm^2$ depending on the number of cells contained within a suitable sub-population. These cells were boiled at 95°C for 10 min and then incubated for 2 h at 65°C in a conventional PCR buffer containing Proteinase K at a final concentration of 1000 $\mu g\ ml^{-1}$, followed by heat inactivation at 85°C for 15 min. Aliquots of this digest solution provided the target DNA template for universal amplification.

### Universal DNA amplification

Duplicate universal amplification reactions were performed for each specimen under paraffin oil in 60 μl volumes containing 1 × Stoffel buffer (Perkin-Elmer, Norwalk, CT, USA), 200 pmols of random 12-mer oligonucleotide, 10 U AmpliTaq DNA Polymerase, Stoffel Fragment (Perkin-Elmer), 200 μM of each dNTP, 5 mM $MgCl_2$, and DNA template. Templates were 10 μl of the digest solution of cells dissected from paraffin tissue sections. Water blanks were included as contamination checks for each amplification series. Thermal cycling conditions were as follows; 92°C (4 min), 40 cycles of 92°C (1 min), 25°C (2 min), 30°C (30 s), 35°C (30 s), 40°C (30 s) and 72°C (2 min) followed by a final extension step of 72°C (15 min). The thermal ramping rate was set at 0.25°C $s^{-1}$ between the 25°C and 30°C steps. A slow ramp rate at low annealing temperature was used to enhance annealing of oligonucleotides to the template DNA. All thermal cycling was performed in a Corbett Research (Sydney, NSW, Australia) FTS-960 Thermal Sequencer (96-well plate format). The duplicate universal amplification products were pooled for each specimen prior to assaying for LOH.

### Loss of heterozygosity assay

LOH was determined using PCR to identify polymorphisms in microsatellite regions of patient constitutional and tumour DNA at a given locus. Aliquots of the pooled universal amplification products generated from DNA isolated from sub-populations of SE, NSGCT, CIS and constitutional control DNA were amplified by PCR using specific oligonucleotide primers flanking each microsatellite marker loci. PCR was performed in 10 μl volumes containing 0.4 U AmpliTaq DNA polymerase

 © 2000 Cancer Research Campaign

(Perkin-Elmer), 10 pmols of each oligonucleotide, 1 μCi [$\alpha$-$^{32}$P] dCTP (3000 Ci mmol$^{-1}$), 20 μM dNTPs, 1 × PCR buffer (Perkin-Elmer) and either 1 μl of universal amplification product or pre-amplified water blank as a contamination check. DNA and control samples were amplified using general thermal cycling conditions as follows; 92°C (3 min), 30 cycles of 92°C (1 min), 48°C (10 s), 50°C (20 s), 54°C (20 s), 58°C (20 s), 62°C (10 s) and 72°C (2 min) followed by a final extension step of 72°C (15 min). All microsatellite repeat markers were obtained from Research Genetics (Huntsville, AL, USA). Differences in the number of repeat sequences between PCR product alleles were resolved using denaturing 7%, 19:1 polyacrylamide gel electrophoresis, with the amplified PCR products being detected via autoradiography. Constitutional DNA was required for LOH analysis to assess normal allele status at any given locus (i.e. heterozygosity or homozygosity). Allelic imbalances were scored visually by comparison of allele intensity in heterozygote patients with obvious reduction of one allele in tumour DNA compared with the intensity of constitutional alleles. Densitometric analysis of typical examples of allelic imbalance showed that alleles with less than 50% reduction in band intensity had been conservatively scored as retaining heterozygosity. After standardizing the tumours against their respective paired constitutive controls allelic imbalance was able to be interpreted as LOH.

Deletion of a specific chromosomal region was defined by the observation of LOH at one or more microsatellite loci located at these chromosomal regions with the following conditions. The observation of LOH at a single microsatellite locus was not deemed sufficient to score as a deletion without further evidence of chromosomal arm monosomy of LOH at adjacent loci. Only a single round of microsatellite PCR was performed for samples showing evidence of LOH as the deletion mapping approach we have used reduces the reliance upon a single result and hence decreases the chance of a false interpretation of allelic imbalance at any specific chromosomal region. All samples were scored for LOH without knowledge of the map position of the microsatellite locus being assessed.

### RESULTS

### Universal amplification of tumour samples

Universal amplification was performed on 36 sub-populations of CIS cells and 87 TGCT specimens comprising 39 seminomas, 13 teratomas, 17 embryonal carcinomas, 12 yolk sac and six mixed NSGCTs isolated from single tumour sections from 40 patients. These included specimens from 24 patients whose tumours represented the pathological continuum observed in TGCTs, including two patients with a combined tumour histology. Multiple specimens of CIS and TGCT cells of varied histology were able to be microdissected from each of these 24 individual patients.

Multiple (~100) microsatellite markers could be reliably amplified from the universal amplification products generated by this modified protocol, without the difficulties generally associated with the PCR analysis of DNA isolated directly from paraffin-embedded tissue. The efficiency of this universal amplification schedule was conservatively estimated at 100-fold amplification. There was no evidence of preferential allelic amplification observed in the constitutional control samples.

### Investigation of deletion at specific chromosomal regions

A panel of 77 microsatellite markers was used to assay the pre-amplified samples for LOH by PCR at chromosomal regions mapping to 3q, 5q, 9p, 11p, 11q, 12q, 17p and 18q (Figure 1). Allele status was assessed at a minimum of three and a maximum of 25 polymorphic loci for each chromosomal region (Table 1). Among these, only four microsatellite loci (D12S64, D17S946, D17S393 and IGF1) demonstrated the retention of heterozygosity for all informative tumour specimens. Comparison of the extent of genetic loss indicated that NSGCTs to have a higher fractional allelic loss (FAL) (mean 0.23) than SE (mean 0.17). There were no significant differences between the mean FAL observed for each NSGCT histological sub-type. LOH was the most frequent for microsatellite loci mapping to chromosome 9. The pattern of allelic loss for several tumours indicated the presence of chromosomal arm monosomy or large deletions affecting 11p (4%), 5q (10%), 3q (11%), 18q (10%) and 9p (23%). Monosomy was not observed in the chromosomal arms 11q, 12q or 17p in any of the tumours examined.

Chromosomal deletion maps were constructed from the pattern of microsatellite allelic losses observed in each TGCT patient. These pre-amplified specimens were assessed for the presence of allelic loss at commonly deleted chromosomal regions located to 3q27–q28, 5q31, 5q34–q35, 9p22–p21, 11p15.5, 11p13, 11q13, 12q22, 17p13 and 18q21.1. A deletion was defined by the observation of LOH at one or more microsatellite loci located at these chromosomal regions. The observation of LOH at a single microsatellite locus was not deemed sufficient to score as a deletion without further evidence of chromosomal arm monosomy or LOH at adjacent loci. The incidence of deletion at each of these chromosomal regions is summarized in Table 2. Losses were noted at every chromosomal region in SE and for all regions except 11q13 in NSGCTs. None of these chromosomal regions

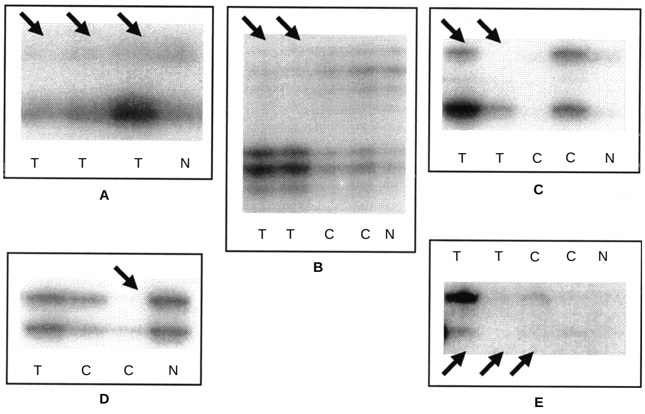

**Figure 1** Illustration of representative loss of heterozygosity (LOH) observed at various microsatellite loci in. TGCTs and CIS specimens. (**A**) LOH at D5S1475 in three tumour specimens; LOH at D9S162 (**B**) and D5S1456 (**C**) in two tumour specimens with retention of heterozygosity in the associated CIS; (**D**) LOH at D5S1456 in the second CIS specimen only with retention of heterozygosity in the tumour and other CIS specimen; (**E**) LOH at D12S393 in two tumours and the first CIS specimen with retention of heterozgosity in the second CIS. Low intensity bands in alleles denoted as demonstrating LOH were attributed to the presence of constitutional DNA contaminating the tumour DNA specimen (C = carcinoma in situ; N = normal control; T = tumour. Arrows indicate alleles demonstrating LOH)

© 2000 Cancer Research Campaign

**Table 1** Summary of percentage LOH observed in TGCTs at individual microsatellite loci

| Locus | Location | Heterozygous tumours (*n*) | Percentage LOH | Locus | Location | Heterozygous tumours (*n*) | Percentage LOH |
|---|---|---|---|---|---|---|---|
| D3S1268 | 3q25.2–q26.2 | 48 | 19 | **D11S922** | **11p15.5** | **46** | **15** |
| D3S1262 | 3q27 | 42 | 26 | **TH** | **11p15.5** | **23** | **13** |
| **D3S1288** | **3q27** | **36** | **31** | **D11S988** | **11p15.5** | **46** | **7** |
| **D3S1265** | **3q27–qter** | 50 | 38 | D11S926 | 11p15.4 | 26 | 12 |
| D3S1272 | 3q27–qter | 29 | 31 | **D11S915** | **11p15.1–p14.1** | **28** | **18** |
| D3S1311 | 3q27–qter | 42 | 21 | **D11S914** | **11p13** | **14** | **29** |
| | | | | D11S907 | 11p13.3 | 41 | 7 |
| D5S421 | 5q22–q23 | 60 | 17 | **FGF3** | **11q13.3** | **38** | **13** |
| D5S622 | 5q23–q31 | 22 | 14 | **INT2** | **11q13.3** | **29** | **3** |
| **D5S393** | **5q31** | **39** | **33** | **D11S911** | **11q13–q23** | **26** | **4** |
| **D5S402** | **5q** | **31** | **16** | – | – | – | – |
| **CSF1R-T** | **5q31–q33.2** | **40** | **23** | D12S64 | – | 49 | 0 |
| **CSF1R** | **5q31–q33.2** | **27** | **7** | D12S82 | 12q21–q22 | 50 | 6 |
| D5S209 | 5q | 21 | 10 | D12S95 | 12q | 47 | 2 |
| D5S412 | 5q32–q33 | 42 | 19 | D12S377 | 12q | 21 | 5 |
| D5S378 | 5q33 | 47 | 26 | **D12S58** | **12q22** | **49** | **12** |
| D5S529 | 5q34 | 54 | 30 | **D12S393** | **12q22** | **45** | **38** |
| D5S621 | 5q34 | 30 | 10 | **D12S296** | **12q22** | **41** | **32** |
| D5S415 | 5q34 | 42 | 15 | **D12S1074** | **12q22** | **38** | **29** |
| D5S671 | 5q34–q35 | 42 | 33 | **D12S346** | **12q22** | **56** | **13** |
| D5S1713 | 5q34–q35 | 41 | 27 | IGF1 | – | 15 | 0 |
| D5S1475 | 5q34–q45 | 48 | 23 | D12S78 | 12q23 | 39 | 15 |
| **D5S1456** | **5q34–q35** | **45** | **33** | D12S84 | 12q23 | 40 | 13 |
| **D5S1402** | **5q34–q35** | **50** | **42** | D12S79 | 12q22–qter | 46 | 13 |
| D5S805 | 5q34–q35 | 43 | 16 | D12S378 | 12q23 | 54 | 24 |
| D5S504 | 5q34–q35 | 38 | 26 | D12S63 | – | 50 | 18 |
| D5S400 | 5q34–q35 | 22 | 5 | D12S367 | 12q–qter | 46 | 7 |
| D5S429 | 5q35 | 30 | 23 | – | – | – | – |
| D5S498 | 5q35 | 51 | 16 | **D17S1289** | **17p13** | **49** | **20** |
| D5S211 | 5q35 | 39 | 15 | **TP53** | **17p13** | **35** | **17** |
| D5S408 | 5q35 | 53 | 26 | D17S793 | – | 33 | 0 |
| GABRA1 | 5q35–qter | 55 | 27 | D17S946 | – | 22 | 0 |
| D9S168 | 9p24–p22 | 57 | 54 | D18S65 | 18q12.2–q12.3 | 39 | 15 |
| D9S156 | 9p23–p22 | 39 | 26 | **D18S46** | **18q21.1** | **26** | **19** |
| D9S268 | 9p23–p22 | 33 | 27 | **DCC** | **18q21.1** | **23** | **39** |
| **D9S285** | **9p23–p22** | **48** | **46** | D18S64 | 18q21.32 | 44 | 25 |
| **D9S162** | **9p22–p21** | **49** | **37** | D18S61 | 18q22.3 | 49 | 12 |
| **D9S171** | **9p21** | **36** | **42** | D18S554 | 18q22.1–qter | 57 | 19 |
| D9S161 | 9p21 | 19 | 21 | D18S70 | 18q23 | 71 | 25 |
| D9S104 | 9p21 | 35 | 31 | – | – | – | – |

Chromosomal regions and microsatellite markers defining regions of common deletion indicated in **bold** typeface. The relative microsatellite marker cytogenetic locations and map positions were deduced using the GDB (http://www.gdb.org), GeneMap 99 (http://www.ncbi.nlm.nih.gov/genemap) and CHLC linkage groupings by interval distribution (http://www.chlc.org)

**Table 2** Regions of common deletion defined by patterns of microsatellite allelic loss

| Defined region | Flanking markers | Tumours showing 1 or more losses at this region (%) | Non-seminoma showing loss (%) | Seminoma showing loss (%) | CIS/ NSGCT showing loss[a] (%) | CIS/ SE showing loss[b] (%) |
|---|---|---|---|---|---|---|
| 3q27–q28 | D3S1288–D3S1265 | 44 | 58 | 33 | 8 | 0 |
| 5q31 | D5S393–CSF1R-T | 29 | 23 | 34 | 13 | 6 |
| 5q34–q35 | D5S1456–D5S1402 | 45 | 53 | 36 | 19 | 7 |
| 9p22–p21 | D9S285–D9S171 | 51 | 64 | 38 | 15 | 13 |
| 11p15.5 | D11S922–D11S988 | 13 | 13 | 13 | 0 | 0 |
| 11p13 | D11S907–D11S914 | 18 | 21 | 15 | 0 | 10 |
| 11q13 | FGF3–INT2 | 4 | 0 | 8 | 0 | 0 |
| 12q22 | D12S58–D12S346 | 44 | 66 | 25 | 43 | 25 |
| 17p13 | D17S1289–TP53 | 22 | 25 | 19 | 0 | 0 |
| 18q21.1 | D18S46–DCC | 46 | 41 | 48 | 0 | 0 |

[a]CIS cells from NSGCT [b]CIS cells from SE

 *© 2000 Cancer Research Campaign*

**Table 3** Summary of allelic losses from individual tumours showing loss at 3q27–q28 in embryonal carcinoma and associated TGCT and CIS specimens

| Tumour histology | Result for each chromosomal region | | | | | | | | | |
|---|---|---|---|---|---|---|---|---|---|---|
| | 3q27–q28 | 5q31 | 5q34–q35 | 9p22–p21 | 11p15 | 11p13 | 11q13 | 12q22 | 17p13 | 18q21 |
| EC | ■ | ○ | ○ | ■ | ○ | ○ | ○ | ■ | ○ | ■ |
| SE | ■ | ○ | ○ | ■ | ○ | ○ | ○ | ■ | ○ | ■ |
| SE | ■ | ○ | ○ | ■ | ○ | ○ | ○ | ○ | ○ | ■ |
| SE | – | ○ | ○ | ■ | ○ | ○ | ○ | ○ | ○ | ■ |
| CIS | ○ | ○ | ○ | ■ | ○ | ○ | ○ | ○ | ○ | ○ |
| CIS | – | ○ | ○ | ○ | ○ | ○ | ○ | ○ | ○ | ○ |
| EC | ■ | ○ | ■ | ■ | ○ | ○ | ○ | ○ | ○ | – |
| EC | ■ | ○ | ■ | ■ | ○ | ○ | ○ | ○ | ○ | – |
| EC | ■ | ○ | ■ | ○ | ○ | ○ | ○ | ○ | ○ | ■ |
| EC | ■ | ○ | ○ | ○ | ○ | ○ | ○ | ○ | ○ | ■ |
| SE | ○ | ○ | ○ | ○ | ○ | ○ | ○ | ○ | ○ | ■ |
| CIS | ○ | ○ | ○ | ○ | ○ | ○ | ○ | ○ | ○ | – |
| CIS | ○ | ○ | ○ | ○ | ○ | ○ | ○ | ○ | ○ | – |
| EC | ■ | ■ | ■ | ■ | ○ | ■ | ○ | ■ | ■ | ○ |
| CIS | ○ | ○ | ○ | ○ | ○ | ○ | ○ | ○ | ○ | ○ |
| EC | ■ | ○ | ○ | ■ | ○ | ■ | ○ | ■ | ○ | ○ |
| CIS | ○ | ○ | ○ | ○ | ○ | ○ | ○ | ○ | ○ | ○ |
| TE | ■ | ○ | ■ | ■ | ○ | ○ | ○ | ■ | ○ | – |
| EC | ■ | ○ | ○ | ■ | ○ | ○ | ○ | ■ | ○ | – |
| EC | ■ | ○ | ○ | ■ | ○ | ○ | ○ | ■ | ○ | – |
| EC | ■ | ○ | ○ | ■ | ○ | ○ | ○ | – | ○ | – |
| YST | ■ | ○ | ○ | ■ | ○ | ○ | ○ | ○ | ○ | – |

■ = allelic deletion; ○ = retention of heterozygosity; – = uninformative at this chromosomal region (homozygosity, no result or not done); CIS = carcinoma in situ; EC = embryonal carcinoma; SE = seminoma; TE = teratoma; YST = yolk-sac tumour. Specimens grouped by tumour histology and CIS sub-populations isolated from individual TGCTs with allelic losses at 3q27–q28 in EC

demonstrated preferential deletion in either SE or NSGCT (Table 2). However, loss of one or more microsatellite loci mapping to 3q27–q28 was observed in all 10 embryonal carcinoma specimens (Table 3), while teratomas and yolk-sac tumours each exhibited loss of this region in only two of six cases. Some heterogeneity in the observed regional chromosome losses was evident between the sub-populations of cell isolates from patients with multiple specimens of CIS (mean 13%), SE (mean 8%) or NSGCT (mean 9%).

Deletion of at least one of these chromosomal regions was observed for every TGCT, with the exception of a single SE (data not shown). The four SE tumour specimens and corresponding CIS specimen isolated from this TGCT showed retention of heterozygosity at all informative regions. Deletion of one or more of the chromosomal regions 3q27–q28, 5q31, 5q34–q35, 9p22–p21, 11p13 and 12q22 was observed in the CIS specimens of several patients representing the pathological continuum observed in TGCTs. The allelotype of deletions observed in both the CIS specimens and associated TGCT components of these patients are summarized in Table 4. The individual components of the two TGCTs with a combined histology demonstrated similar patterns of allelic loss at multiple chromosomal regions (Table 5). Allelic deletion at chromosomal region 9p21 was observed in a single CIS specimen and in both the NSGCT and SE tumour components of the first combined tumour. All of these TGCT specimens showed additional losses of 18q21 as well as an accumulation of deletions at 3q27–q28 and 12q22. There was only a single loss noted at 18q21 in the SE component of the second combined tumour and no losses in either of the associated CIS specimens. All four of the NSGCT components for this combined histology TGCT demonstrated loss of 3q27–q28 in addition to deletion at other chromosomal regions including 18q21.

### DISCUSSION

The ability to assay specific sub-populations of enriched TGCT and CIS cells for LOH at multiple sites provides a valuable allelotyping tool for determining clonal relationships and for identifying any critical losses required for tumour progression. This study is the first to investigate the early molecular genetic changes in CIS and the associated SE and NSGCT cells. The universal amplification protocol we have used to achieve this has potential errors with regard to preferential allelic amplification generating false-positive LOH results in the pre-amplified DNA specimens. However, previous experiments have demonstrated that universal amplification products are an accurate representation of the allelic ratio of the original DNA sample, since no evidence of preferential allelic amplification was observed (Faulkner and Leigh, 1998). Recently, the accuracy of this approach has been confirmed by others (Paulson et al, 1999). In addition, the comparison of alleles from the pre-amplified tumour specimens with those of the pre-amplified constitutional control samples, which did not demonstrate preferential allelic amplification, further minimizes the possibility of false-positive LOH results being generated by universal amplification.

In this study of TGCTs, a high degree of genetic loss was evident at many sites throughout the genome. Our results identify a novel site of frequent deletion at 5q31 and confirm the results of previous investigations noting regions of common deletion in TGCTs at 3q27–28, 5q34–q35, 9p22–p21, 11p15.5, 11p13, 11q, 12q22, 17p13 (TP53) and 18q21.1 (DCC) (Lothe et al, 1993; Murty et al, 1994*a* 1994*b*; 1996*a*; 1996*b*; Peng et al, 1995; International Testicular Cancer Linkage Consortium, 1998). The 5q31 chromosomal region has previously been implicated in

*© 2000 Cancer Research Campaign*

**Table 4** Summary of allelic losses from individual tumours showing losses in CIS and associated TGCT specimens

| Tumour histology | 3q27–q28 | 5q31 | 5q34–q35 | 9p22–p21 | 11p15 | 11q13 | 11p13 | 12q22 | 17p13 | 18q21 |
|---|---|---|---|---|---|---|---|---|---|---|
| TE | – | ○ | ■ | ○ | – | – | – | ■ | – | – |
| TE | – | ○ | ■ | ○ | – | – | – | ○ | ○ | – |
| EC | – | ○ | ■ | ○ | – | – | – | ■ | ○ | – |
| EC | – | ○ | ■ | ○ | – | – | – | ○ | ○ | – |
| YST | – | ○ | ■ | ○ | – | – | – | ■ | ○ | – |
| NSGCT | ○ | ■ | ■ | ○ | – | – | – | ■ | ○ | – |
| CIS | ○ | ○ | ■ | ○ | – | – | – | ■ | ○ | – |
| CIS | ○ | ■ | ■ | ○ | – | – | – | ○ | ○ | – |
| CIS | ○ | ○ | ○ | ○ | – | – | – | ■ | ○ | – |
| CIS | ○ | ○ | ○ | ○ | – | – | – | ■ | ○ | – |
| TE | ○ | ○ | ○ | ■ | ■ | ■ | – | ■ | ■ | ■ |
| YST | ○ | ○ | ○ | ■ | ■ | ■ | – | ■ | ■ | ■ |
| CIS | ○ | ○ | ○ | ■ | ○ | ○ | – | ■ | ○ | ○ |
| CIS | ○ | ○ | ○ | – | ○ | ○ | – | ■ | ○ | ○ |
| SE | ○ | ■ | ○ | ■ | ○ | ○ | – | ○ | – | ○ |
| SE | ○ | ■ | ○ | ■ | ○ | – | – | ○ | ○ | ○ |
| CIS | ○ | ■ | ○ | ■ | ○ | ○ | – | ○ | ○ | ○ |
| SE | ○ | ○ | ○ | ○ | ○ | ○ | ○ | ■ | ○ | ○ |
| SE | ○ | ○ | ○ | ○ | ○ | ○ | ○ | ○ | ○ | ○ |
| CIS | ○ | ○ | ○ | ○ | ○ | ○ | ○ | ○ | ○ | ○ |
| CIS | ○ | ○ | ○ | ○ | ○ | ○ | ○ | ■ | ○ | ○ |
| CIS | ○ | ○ | ○ | ○ | ○ | ○ | ○ | ■ | ○ | ○ |
| SE | ■ | ○ | ■ | ○ | ○ | ○ | – | ■ | ○ | ■ |
| SE | ■ | ○ | ■ | ○ | ○ | ○ | – | ■ | ○ | ■ |
| CIS | ○ | ○ | ○ | ○ | ○ | ○ | – | ■ | ○ | ○ |
| SE | ○ | ■ | ■ | ■ | ○ | – | ■ | ■ | ○ | ■ |
| SE | ○ | ■ | ■ | ■ | ○ | – | ■ | ■ | ○ | ■ |
| CIS | ○ | ○ | ○ | ○ | ○ | – | ○ | ○ | ○ | ○ |
| CIS | ○ | ○ | ○ | ○ | ○ | – | ○ | ■ | – | ○ |
| TE | ■ | ■ | ■ | ■ | ○ | – | ○ | ■ | ■ | – |
| YST | ■ | ■ | ■ | ■ | ○ | – | ○ | ■ | ■ | – |
| CIS | ■ | ○ | ○ | – | ○ | – | ○ | ■ | ○ | – |
| CIS | – | ○ | ○ | – | ○ | – | ○ | ■ | ○ | – |
| SE | ○ | ○ | ○ | ○ | ○ | ■ | – | ○ | – | ○ |
| SE | ○ | ○ | ○ | ○ | ○ | ■ | – | ○ | ○ | ○ |
| CIS | ○ | ○ | ○ | ○ | ○ | ■ | ○ | ○ | ○ | ○ |
| NSGCT | ○ | ■ | ○ | ○ | ■ | ○ | ○ | ■ | ○ | ○ |
| CIS | ○ | ■ | ○ | ○ | ○ | ○ | ○ | ○ | ○ | ○ |
| CIS | ○ | ○ | ■ | ■ | ○ | ○ | ○ | ○ | ○ | ○ |
| SE | ○ | ■ | ○ | ■ | ○ | – | ○ | ■ | ■ | ○ |
| SE | ○ | ■ | ○ | ○ | ○ | – | ○ | ■ | ■ | ○ |
| SE | – | ○ | ○ | ■ | ○ | – | ○ | ○ | ○ | – |
| CIS | ○ | ○ | ○ | ○ | ○ | – | ○ | ○ | ○ | ○ |
| CIS | ○ | ○ | ■ | ○ | ○ | – | ○ | ○ | ○ | – |

■ = allelic deletion; ○ = retention of heterozygosity; – = uninformative at this chromosomal region (homozygosity, no result or not done); CIS = carcinoma in situ; EC = embryonal carcinoma; NSGCT = mixed NSGCT; SE = seminoma; TE = teratoma; YST = yolk-sac tumour. Specimens grouped by tumour histology and CIS sub-populations isolated from individual patients with allelic losses in CIS

TGCTs due to synteny with the murine chromosome 18 where the teratoma susceptibility gene (*Ter*) has been mapped (Asada et al, 1994). More importantly, deletion of 3q27–q28, 5q31, 5q34–q35, 9p22–p21, 11p13 and 12q22 was also noted in CIS cells, which has not been previously described.

Considering the high frequency of loss and evidence of losses in CIS, the regions of loss observed at 3q27–q28, 5q31, 5q34–q35, 9p22–p21 and 12q22 are of particular interest. Losses of all five noted regions were observed in all histological subtypes, suggesting an important role for potential genes located at these sites in the development of male GCTs. Our results suggest that loss or inactivation of gene(s) located within the 3q27–q28 deleted region appears essential for the development of embryonal carcinoma while loss of this region is not required for the development of teratoma or yolk-sac tumour (Table 3). Familial linkage studies have previously identified 3q27–q28 as a candidate region containing putative gene(s) involved in the development of TGCTs (International Testicular Cancer Linkage Consortium, 1998). Loss of these putative genes may contribute to the inability of these pluripotent embryonal carcinoma cells to differentiate and thereby represent the formation of pure null-potent embryonal carcinoma. Taken together these findings may represent an important step in the identification of critical genes involved in the development of TGCTs and more specifically the development of embryonal carcinoma.

A degree of heterogeneity was evident in the chromosomal losses identified between sub-populations of cells for the patients with multiple specimens of CIS, SE or NSGCT. One possible explanation for this observation is that several genes may be involved in a multi-step mechanism of carcinogenesis and clonal reprogramming acting in both CIS and TGCTs. There were no data to suggest that loss of one or more specific regions in either CIS or

 *© 2000 Cancer Research Campaign*

**Table 5** Summary of deletion allelotype for the CIS, SE and NSGCT components of TGCT in combined histology tumours

| Tumour histology | Result for each chromosomal region | | | | | | | | | |
|---|---|---|---|---|---|---|---|---|---|---|
| | 3q27–q28 | 5q31 | 5q34–q35 | 9p22–p21 | 11p15 | 11p13 | 11q13 | 12q22 | 17p13 | 18q21 |
| EC | ■ | ○ | ○ | ■ | ○ | ○ | ○ | ■ | ○ | ■ |
| SE | ■ | ○ | ○ | ■ | ○ | ○ | ○ | ■ | ○ | ■ |
| SE | ■ | ○ | ○ | ■ | ○ | ○ | ○ | ○ | ○ | ■ |
| SE | – | ○ | ○ | ■ | ○ | ○ | ○ | ○ | ○ | ■ |
| CIS | ○ | ○ | ○ | ■ | ○ | ○ | ○ | ○ | ○ | ○ |
| CIS | – | ○ | ○ | ○ | ○ | ○ | ○ | ○ | ○ | ○ |
| EC | ■ | ○ | ■ | ■ | ○ | ○ | ○ | ○ | ○ | – |
| EC | ■ | ○ | ■ | ■ | ○ | ○ | ○ | ○ | ○ | – |
| EC | ■ | ○ | ■ | ○ | ○ | ○ | ○ | ○ | ○ | ■ |
| EC | ■ | ○ | ○ | ○ | ○ | ○ | ○ | ○ | ○ | ■ |
| SE | ○ | ○ | ○ | ○ | ○ | ○ | ○ | ○ | ○ | ■ |
| CIS | ○ | ○ | ○ | ○ | ○ | ○ | ○ | ○ | ○ | – |
| CIS | ○ | ○ | ○ | ○ | ○ | ○ | ○ | ○ | ○ | – |

■ = allelic deletion; ○ = retention of heterozygosity; – = uninformative at this chromosomal region (homozygosity, no result or not done); CIS = carcinoma in situ; EC = embryonal carcinoma; SE = seminoma. Specimens grouped by tumour histology and CIS sub-populations isolated from individual patients with a TGCT with the histology of a combined tumour invasive tumour is required for the preferential development of either SE or NSGCT, with the noted exception of 3q27–q28 in embryonal carcinoma. These data support the previously proposed model for a single neoplastic pathway of progression from CIS precursor cells to NSGCT with SE as an intermediate stage of development (Ewing, 1911; Friedman, 1951; Raghavan et al, 1982; Oliver et al, 1987; Oosterhuis et al, 1989).

The inability of SE to express embryonic differentiation suggests that specific chromosomal regions retained in these tumours may contain genes regulating gonocytic differentiation. A marked increase in loss at both 9p22–p21 and 12q22 was observed in NSGCTs compared to SE. Deletion or inactivation of genes located at these regions could represent loss of negative regulatory elements for the expression of embryonic differentiation. The absence of allelic losses in CIS for either TP53 or DCC suggests that loss of these genes may be a late event involved in the progression and clonal growth of an invasive tumour, rather than an early event involved in the carcinogenesis of TGCTs. Chromosome deletions of 17p and 18q have been well characterized as late events in the development of colorectal carcinoma (Vogelstein et al, 1988).

DNA flow cytometry has shown that NSGCTs have a lower DNA ploidy value than SE, leading to speculation that tumour progression in TGCTs may be associated with a net non-random loss of chromosomes in SE leading to NSGCT (Oosterhuis et al, 1989; Fosså et al, 1991; De Graaf et al, 1992). We have observed a higher mean fractional allelic loss (FAL) for NSGCT when compared to SE, which is consistent with these observations of chromosome loss in NSGCT. Unlike a previous study, we find no significant difference between the FAL for teratomas when compared to embryonal carcinoma (Murty et al, 1994*a*).

The pathogenetic relationships between the subtypes of TGCTs were explored by an allelotype analysis of the chromosomal deletions in CIS and the associated SE or NSGCT cells. Assessment of the clonal relationships between the specimens of CIS and TGCT elements for the combined histology tumours provided further evidence for the clonal 'reprogramming' of SE into NSGCT by the accumulation of genetic losses as outlined by the linear progression model (Ewing, 1911; Friedman, 1951; Raghavan et al, 1982; Oliver et al, 1987; Oosterhuis et al, 1989) (Table 5). This clonal relationship could be traced directly from the different CIS and SE specimens through to NSGCT (EC) in the first case of combined tumour. The tumour components of the second combined histology TGCT also appeared to be derived from a monoclonal origin. Although no losses were observed in either of the CIS specimens for this TGCT, there was evidence of clonal 'reprogramming' occurring in the SE specimen to the more advanced, though related, NSGCT (EC) components. Combined tumours may therefore represent heterogeneous sub-populations of SE and NSGCT cells progressing from related CIS clones at various stages of progression through a single neoplastic pathway.

Out data has demonstrated a variety of allelic losses in the sub-populations of CIS and TGCT cells, including deletions in sub-populations of CIS or TGCT not observed in the other associated TGCT cells (Table 4). This tumour heterogeneity might also be considered as evidence of clonal progression occurring within the different regions of the tumour. The observation of this phenomenon in CIS suggests that clonal progression is occurring even at this early stage of carcinogenesis. These data support the previously proposed model that sub-populations of CIS cells may accumulate mitotic errors in multiple genes that represent the unknown genetic switch to develop into an invasive tumour, which may represent the formation of SE. Alternatively, some CIS may undergo clonal 'reprogramming' by loss or inactivation of the critical genes responsible for the maintenance of gonocytic differentiation. These CIS may then evolve directly into NSGCT following the initiation event, thereby bypassing the SE intermediate (for review, see Oosterhuis and Looijenga, 1993). The increased loss of 12q22 we have observed in CIS from NSGCTs compared to CIS from their SE counterparts may represent the loss of such critical regulatory genes residing within this chromosomal region. Evidence for the under-representation of chromosome 12 and 15 in CIS adjacent to NSGCT when compared to CIS adjacent to SE has previously demonstrated the progressive karyotypic evolution of CIS by chromosome loss (Looijenga et al, 1993; Gillis et al, 1994). We speculate that the SEs exhibiting a degree of loss at the commonly deleted regions comparable to NSGCTs may represent highly malignant SE still under gonocytic differentiation control not yet able to 'reprogramme' to an embryonal lineage. The single

© 2000 Cancer Research Campaign

TGCT with no losses may represent a SE at the early stages of clonal progression with low malignant potential.

We conclude that deletion of gene(s) within the regions of common deletion at 12q22, 5q34–q35, 5q31, 3q27–q28 and 9p21–p22 are early events in the carcinogenesis of TGCTs, and that SE is an intermediate stage of development within a single neoplastic pathway of progression from CIS precursor cells to NSGCT. In addition, loss or inactivation of gene(s) located at 3q27–q28 possibly explains the inability of null-potent embryonal carcinoma to differentiate into somatic or extra-embryonic tissue, and deserves further study.

### ACKNOWLEDGEMENTS

We thank Roger Crouch for assistance with the pathological review of the tumour sections.

### REFERENCES

Asada Y, Varnum DS, Frankel WN and Nadeau FJ (1994) A mutation in the Ter gene causing increased susceptibility to testicular teratomas maps to mouse chromosome 18. *Nat Genet* **6**: 263–368

Atkin NB and Baker MC (1982) Specific chromosome change, i(12p), in testicular tumours? *Lancet* **2**: 1349

Batata MA, Chu FC, Hilaris BS, Whitmore WF and Golbey RB (1982) Testicular cancer in cryptorchids. *Cancer* **49**: 1023–1030

Bredael JJ, Vugrin D and Whitmore WJ Jr (1982) Autopsy findings in 154 patients with germ cell tumours of the testis. *Cancer* **50**: 548–551

Casa E and Kirkpatrick BW (1996) Evaluation of different amplification protocols for use in primer-extension preamplification. *Biotechniques* **20**: 219–225

Castedo SMMJ, de Jong B, Oosterhuis JW, Seruca R, te Meerman GJ, Dam A and Schraffordt Koops H (1989*a*) Cytogenetic analysis of ten human seminomas. *Cancer Res* **49**: 439–443

Castedo SMMJ, de Jong B, Oosterhuis JW, Seruca R, Idenburg VJS, Dam A, te Meerman GJ, Schraffordt-Koops H and Sleijfer DT (1989*b*). Chromosomal changes in human primary testicular nonseminomatous germ cell tumours. *Cancer Res* **49**: 5696–5701

Crisan D and Mattson JC (1993) Retrospective DNA analysis using fixed tissue specimens. *DNA Cell Biol* **12**: 455–464

Czaja JT and Ulbright TM (1992) Evidence for the transformation of seminoma to yolk sac tumour, with histogenetic considerations. *Am J Clin Pathol* **97**: 468–477

Damjanov I (1989) Is seminoma a relative or a precursor of embryonal carcinoma? *Lab Invest* **60**: 1–3

De Graaff WE, Oosterhuis JW, de Jong B, Dam A, van Putten WLJ, Castedo SMMJ, Sleijfer DT and Schraffordt-Koops H (1992) Ploidy of testicular carcinoma in situ. *Lab Invest* **66**: 166–168

de Jong B, Oosterhuis JW, Castedo SMMJ, Vos ATM and te Meerman GJ (1990) Pathogenesis of adult testicular germ cell tumours. A cytogenetic model. *Cancer Genet Cytogenet* **48**: 143–167

Ewing J (1911) Teratoma testis and its derivatives. *Surg Gynecol Obstet* **12**: 230–261

Faulkner SW and Leigh DA (1998) Universal amplification of DNA isolated from small regions of paraffin-embedded formalin-fixed tissue. *Biotechniques* **24**: 47–50

Fosså SD, Nesland JM, Pettersen EO, Amellem O, Wæhre H and Heimdal K (1991) DNA ploidy in primary testicular cancer. *Br J Cancer* **64**: 948–952

Friedman NB (1951) The comparative morphogenesis of extragenital and gonadal teratoid tumours. *Cancer* **4**: 265–276

Gillis AJM, Looijenga LHJ, de Jong B and Oosterhuis JW (1994) Conality of combined testicular germ cell tumours of adults. *Lab Invest* **71**: 874–878

International Testicular Cancer Linkage Consortium (1998) Candidate regions for testicular cancer susceptibility genes. *APMIS* **106**: 64–72

Jacobsen GK, Hendriksen OB and von der Maase H (1981) Carcinoma in situ of testicular tissue adjacent to malignant germ cell tumours: a study of 10 cases. *Cancer* **47**: 2660–2662

Kiss F and Jubasz J (1985) Testicular germ cell tumours, current problems of histogenesis and classification. *Int Urol Nephrol* **17**: 85–95

Looijenga LHJ, Gillis AJM, van Putten WLJ and Oosterhuis JW (1993) In situ numeric analysis of centromeric regions of chromosomes 1, 12 and 15 of seminomas, nonseminomatous germ cell tumours, and carcinoma in situ of human testis. *Lab Invest* **68**: 211–219

Lothe RA, Hastie N, Heimadal K, Fosså SD, Stenwig AE and Børresen AL (1993) Frequent loss of 11p13 and 11p15 loci in male germ cell tumours. *Genes Chromosomes Cancer* **7**: 96–101

Mostert MC, Verkerk AJ, van de Pol M, Heighway J, Marynen P, Rosenberg C, van Kessel AG, van Echten J, de Jong B, Oosterhuis JW and Looijenga LH (1998) Identification of the critical region of 12p over-representation in testicular germ cell tumours of adolescents and adults. *Oncogene* **16**: 2617–2627

Mostofi FK (1986) Pathology and germ cell tumours of the testis. *Cancer* **45**: 1735–1754

Mostofi FK and Sobin LH (1977) Histological typing of testis tumours. In: *International histological classification of tumours No. 16* WHO: Geneva.

Murty VVVS, Bosl GJ, Houldsworth J, Meyers M, Mukherjee AB, Reuter V and Chaganti RSK (1994*a*) Allelic loss and somatic differentiation in human male germ cell tumours. *Oncogene* **9**: 2245–2251

Murty VVVS, Li R-G, Houldsworth J, Bronson DL, Reuter VE, Bosl GJ and Chaganti RSK (1994*b*) Frequent allelic deletions and loss of expression characterise the DCC gene in male germ cell tumours. *Oncogene* **9**: 1–5

Murty VVVS, Renault B, Falk CT, Bosl GJ, Kucherlapati R and Chaganti RSK (1996*a*) Physical mapping of a commonly deleted region, the site of a candidate tumour suppressor gene, at 12q22 in human male germ cell tumours. *Genomics* **35**: 562–570

Murty VVVS, Reuter VE, Bosl GJ and Chaganti RSK (1996*b*) Deletion mapping identifies loss of heterozygosity at 5p15.1–p15.2, 5q11 and 5q34–35 in human male germ cell tumours. *Oncogene* **12**: 2719–2723

Oliver RTD (1987) HLA phenotype and clinicopathological behaviour of germ cell tumours: possible evidence for clonal evolution from seminomas to NSGCT. *Int J Androl* **10**: 85–94

Oosterhuis JW and Looijenga LHJ (1993) The biology of human germ cell tumours: retrospective speculations and new prospectives. *Eur Urol* **23**: 245–250

Oosterhuis JW, Castedo SMMJ, de Jong B, Cornelisse CJ, Dam A, Th Sleijfer D and Schraffordt-Koops H (1989) Ploidy of primary germ cell tumours of the testis: pathogenetic and clinical relevance. *Lab Invest* **60**: 14–20

Paulson TG, Galipeau PC and Reid BJ (1999) Loss of heterozygosity analysis using whole genome amplification, cell sorting and fluorescence-based PCR. *Genome Res* **9**: 482–491

Peng H-Q, Bailey D, Bronson D, Goss PE and Hogg D (1995) Loss of heterozygosity of tumour suppressor genes in testis cancer. *Cancer Res* **55**: 2871–2875

Pierce GB and Abell MR (1970) Embryonal carcinoma of the testis. *Pathol Annu* **5**: 27–60

Raghavan D, Sullivan AL, Peckham MJ and Neville AM (1982) Elevated serum alphafetoprotein and seminoma. Clinical evidence for a histologic continuum? *Cancer* **50**: 982–989

Rodriguez E, Houldsworth J, Reuter VE, Meltzer P, Zhang J, Trent JM, Bosl GJ and Chaganti RSK (1993) Molecular cytogenetic analysis of i(12p)-negative human male germ cell tumours. *Genes Chromosomes Cancer* **8**: 230–236

Sesterhenn IA (1985) The role of intratubular malignant germ cells in the histogenesis of germ cell tumours. In: *Germ Cell Tumours II: Proceedings of the Second Germ Cell Tumours Conference*, Jones WG, Milford-Ward A and Anderson CK (eds) pp25–35. Pergamon Press: New York

Shibata D, Hawes D, Li Z-H, Hernandez AM, Spruck CH and Nichols P (1992) Specific genetic analysis of microscopic tissue after selective ultraviolet radiation fractionation and the polymerase chain reaction. *Am J Pathol* **141**: 539–543

Skakkebæk NE, Bethelsen JG, Giwercman A and Muller J (1987) Carcinoma of the testis: possible origin from gonocytes and precursor of all types of germ cell tumours except spermatocytoma. *Int J Androl* **10**: 19–28

Suijkerbuik RF, Sinke RJ, Meloni AM, Parrington JM, van Echten J, de Jong G, Oosterhuis JW, Sandberg AA and Geurts van Kessel A (1993) Over-representation of chromosome 12p sequences and karyotypic evolution in i(12p)-negative testicular germ cell tumours revealed by fluorescence in situ hybridization. *Cancer Genet Cytogenet* **70**: 85–93

van der Maase H, Rorth M, Walbom-Jorgensen S, Sorensen BL, Christophersen IS, Hald T, Jacobsen GK, Bethelsen JG and Skakkebæk NE (1986) Carcinoma in situ of the contralateral testis in patients with testicular germ cell cancer: study of 27 cases in 500 patients. *Br J Med* **293**: 1398–1401

Vogelstein B, Fearon ER, Stanley BA, Hamilton R, Kern SE, Preisinger AC, Leppert M, Nakamura Y, White R, Smits AMM and Bos JL (1988) Genetic alterations during colorectal-tumour development. *N Engl J Med* **319**: 525–532

Zhang L, Cui X, Schmitt K, Hubert R, Navidi W and Arnheim N (1992) Whole genome amplification from a single cell: Implications for genetic analysis. *Proc Natl Acad Sci USA* **89**: 5847–5851

© 2000 Cancer Research Campaign
